# Supplementary material for: Plant Responses of Maize to Two formae speciales of Sporisorium reilianum Support Recent Fungal Host Jump
Source: Int J Mol Sci. 2023 Oct 26;24(21):15604. doi: 10.3390/ijms242115604 (PMC10648682; doi:10.3390/ijms242115604)
Supplement: Supplementary file 1 [file ijms-24-15604-s001.zip › ijms-2674811-supplementary.pdf]

Supplemental Material

Plant responses of maize to two formae speciales of Sporisorium reilianum support recent fungal host jump

Lukas D. Dittiger 1, Shivam Chaudhary 1, Alexandra C. U. Furch 2, Axel Mithöfer 3 and Jan Schirawski 1,*

1 Department of Genetics, Matthias Schleiden Institute, Friedrich Schiller University Jena, Philosophenweg 12, 07743 Jena, Germany; lukas.dorian.dittiger@uni-jena.de; shivam.chaudhary@uni-jena.de; jan.schirawski@uni-jena.de

2 Department of Plant Physiology, Matthias Schleiden Institute, Friedrich Schiller University Jena, Dornburgerstr. 159, 07743 Jena, Germany; alexandra.furch@uni-jena.de

3 Research Group Plant Defense Physiology, Max Planck Institute for Chemical Ecology, Hans-Knöll-Straße 8, 07745 Jena, Germany; amithoefer@ice.mpg.de

* Correspondence: jan.schirawski@uni-jena.de; Tel.: +49-3641-949555

Figure S1

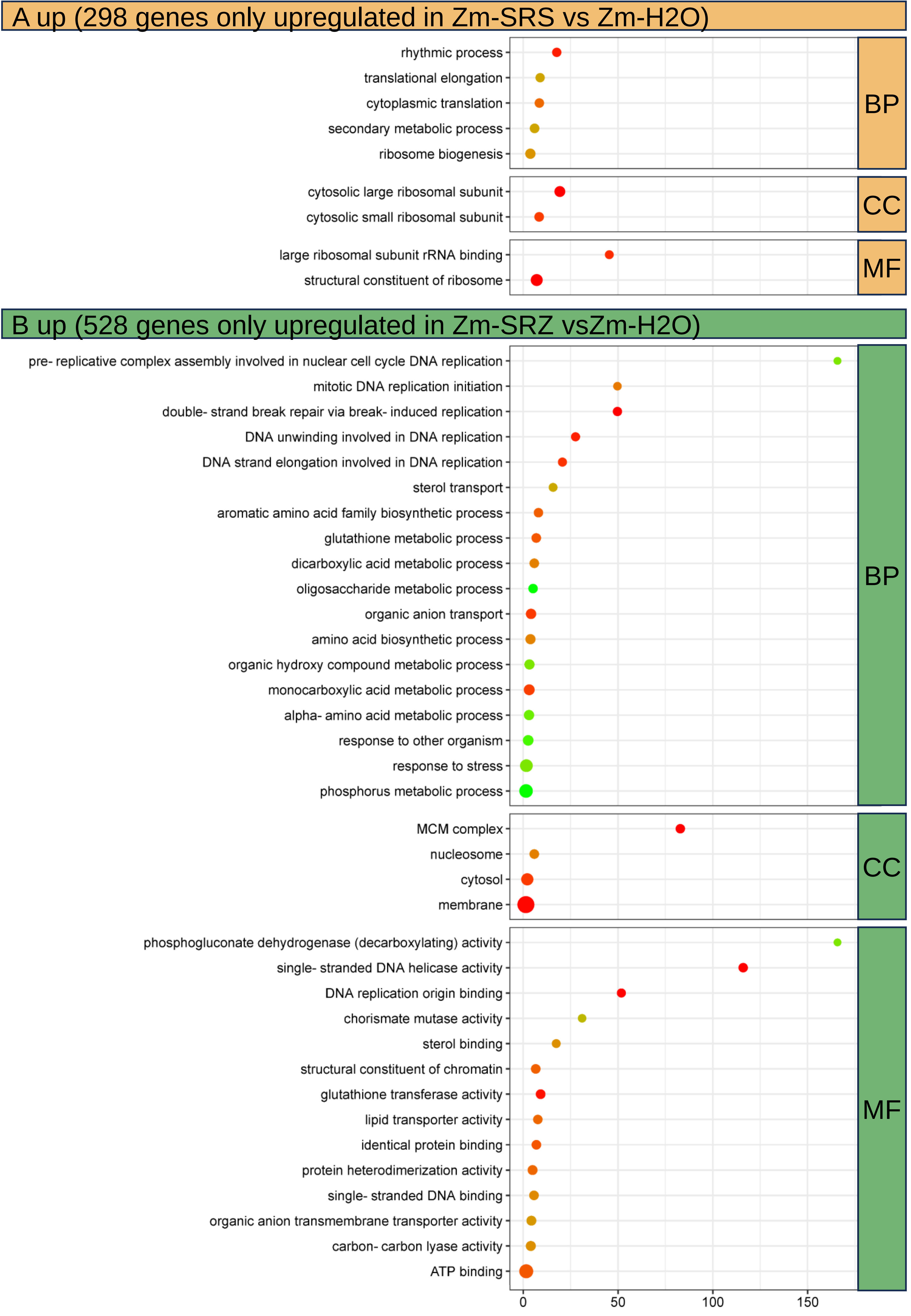

A up (298 genes only upregulated in Zm-SRS vs Zm-H2O)
rhythmic process
translational elongation
cytoplasmic translation
secondary metabolic process
ribosome biogenesis
BP
cytosolic large ribosomal subunit
cytosolic small ribosomal subunit
CC
large ribosomal subunit rRNA binding
structural constituent of ribosome
MF
B up (528 genes only upregulated in Zm-SRZ vsZm-H2O)
pre- replicative complex assembly involved in nuclear cell cycle DNA replication
mitotic DNA replication initiation
double- strand break repair via break- induced replication
DNA unwinding involved in DNA replication
DNA strand elongation involved in DNA replication
sterol transport
aromatic amino acid family biosynthetic process
glutathione metabolic process
dicarboxylic acid metabolic process
oligosaccharide metabolic process
organic anion transport
amino acid biosynthetic process
organic hydroxy compound metabolic process
monocarboxylic acid metabolic process
alpha- amino acid metabolic process
response to other organism
response to stress
phosphorus metabolic process
BP
MCM complex
nucleosome
cytosol
membrane
CC
phosphogluconate dehydrogenase (decarboxylating) activity
single- stranded DNA helicase activity
DNA replication origin binding
chorismate mutase activity
sterol binding
structural constituent of chromatin
glutathione transferase activity
lipid transporter activity
identical protein binding
protein heterodimerization activity
single- stranded DNA binding
organic anion transmembrane transporter activity
carbon- carbon lyase activity
ATP binding
MF
0
50
100
150

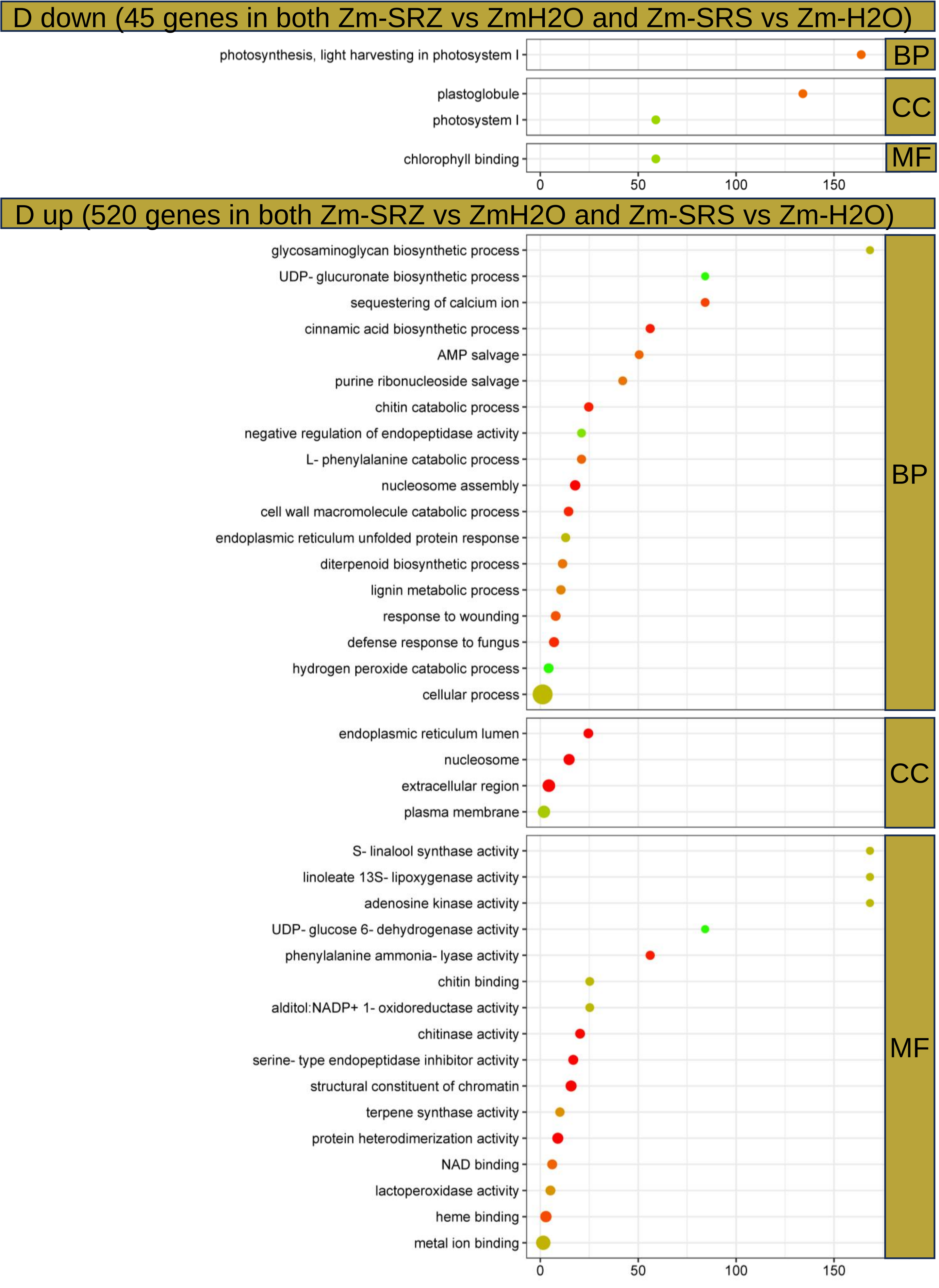
D down (45 genes in both Zm-SRZ vs ZmH2O and Zm-SRS vs Zm-H2O)
photosynthesis, light harvesting in photosystem I
BP
plastoglobule
photosystem I
CC
chlorophyll binding
MF
0
50
100
150
D up (520 genes in both Zm-SRZ vs ZmH2O and Zm-SRS vs Zm-H2O)
glycosaminoglycan biosynthetic process
UDP- glucuronate biosynthetic process
sequestering of calcium ion
cinnamic acid biosynthetic process
AMP salvage
purine ribonucleoside salvage
chitin catabolic process
negative regulation of endopeptidase activity
L- phenylalanine catabolic process
nucleosome assembly
cell wall macromolecule catabolic process
endoplasmic reticulum unfolded protein response
diterpenoid biosynthetic process
lignin metabolic process
response to wounding
defense response to fungus
hydrogen peroxide catabolic process
cellular process
BP
endoplasmic reticulum lumen
nucleosome
extracellular region
plasma membrane
CC
S- linalool synthase activity
linoleate 13S- lipoxygenase activity
adenosine kinase activity
UDP- glucose 6- dehydrogenase activity
phenylalanine ammonia- lyase activity
chitin binding
alditol:NADP+ 1- oxidoreductase activity
chitinase activity
serine- type endopeptidase inhibitor activity
structural constituent of chromatin
terpene synthase activity
protein heterodimerization activity
NAD binding
lactoperoxidase activity
heme binding
metal ion binding
MF
0
50
100
150

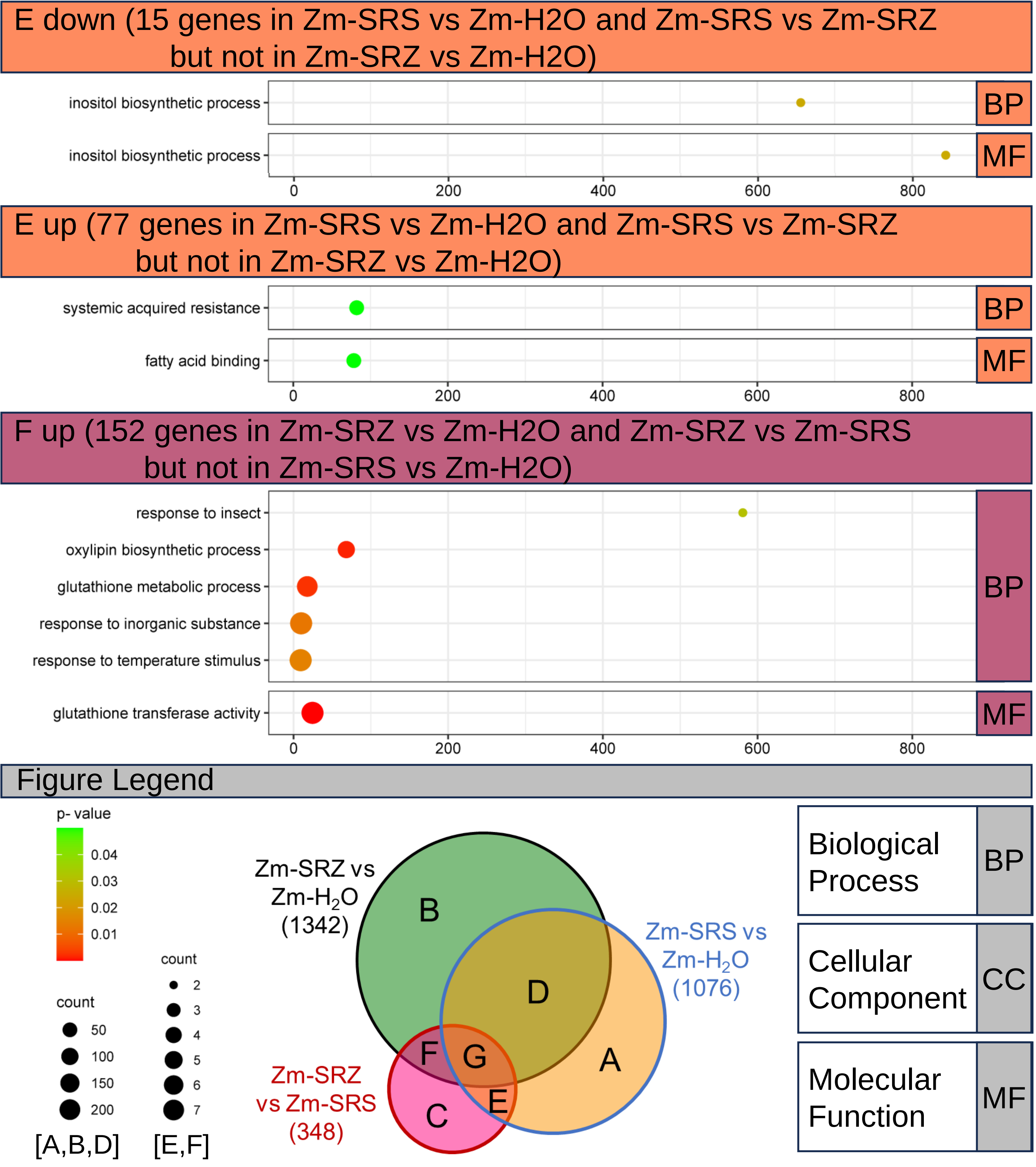

Figure S1. Enrichment analysis of GO terms associated with deregulated maize genes. Only GO terms with adjusted P-values below 0.05 are listed. Genes were analyzed in different sets (A, B, D, E, F) of deregulated genes according to the labeling in the Venn diagram below. For bubble plot size and color explanation see legend below. Bubbles are sorted according to fold enrichment of the specific GO term in the set of analyzed genes relative to in all genes. Missing entries indicate lack of significant GO terms.
